# Conventional Soybean Meal as Fishmeal Alternative in Diets of Japanese Seabass (*Lateolabrax japonicus*): Effects of Functional Additives on Growth, Immunity, Antioxidant Capacity and Disease Resistance

**DOI:** 10.3390/antiox11050951

**Published:** 2022-05-12

**Authors:** Jie Wang, Kangsen Mai, Qinghui Ai

**Affiliations:** 1 Key Laboratory of Aquaculture Nutrition and Feed (Ministry of Agriculture and Rural Affairs), Key Laboratory of Mariculture (Ministry of Education), Ocean University of China, 5 Yushan Road, Qingdao 266003, China; wangjie03@caas.cn (J.W.); kmai@ouc.edu.cn (K.M.); 2 Laboratory for Marine Fisheries Science and Food Production Processes, Qingdao National Laboratory for Marine Science and Technology, 1 Wenhai Road, Qingdao 266237, China; 3 National Aquafeed Safety Assessment Center, Institute of Feed Research, Chinese Academy of Agricultural Sciences, Beijing 100081, China

**Keywords:** *Lateolabrax japonicas*, functional additives, conventional soybean meal, growth performance, immunity, antioxidant capacity, disease resistance

## Abstract

Aiming to optimize soymeal-based diets for Japanese seabass (*Lateolabrax japonicas*), a 105-day feeding trial was conducted to evaluate the effects of functional additives, including antioxidants (ethoxyquin, thymol and carvacrol) and chelated trace elements (Cu, Mn and Zn), on the growth, immunity, antioxidant capacity and disease resistance of fish fed diets with conventional soybean meal replacing 50% of fishmeal. Three isonitrogenous (45%) and isolipidic (11%) diets were formulated: (1) standard reference diet (FM, 42% fishmeal); (2) soymeal-based diet (SBM, 21% fishmeal and 30% conventional soybean meal); (3) SBM diet supplemented 0.0665% functional additives (FAS). Each experimental diet was randomly fed to quadruplicate groups of forty feed-trained Japanese seabass (initial average body weight = 125.6 ± 0.6 g) stocked in a saltwater floating cage. Upon the conclusion of the feeding trial, lower feed intake was observed in fish fed SBM compared to those fed FM and FAS. Fish fed FM showed the highest growth performance, estimated as the weight gain rate. Notably, FAS supported faster growth of fish than those fed SBM, indicating the optimal growth performance of dietary functional additives. The feed conversion rate showed the opposite trend among dietary treatments, with the highest value in fish fed SBM. Regarding immunity, fish fed soymeal-based diets suppressed the serum alternative complement pathway activities compared to FM, whereas the respiratory burst activity in macrophages of head kidneys showed a similar picture, but no statistical differences were observed. Further, fish fed soymeal-based diets had lower serum Cu-Zn SOD, CAT and GPx activities as well as liver vitamin E levels and scavenging rates of hydroxyl radical but higher liver MDA contents compared to the FM-fed group. Fish fed FAS had higher serum Cu-Zn SOD and GPx activities and liver vitamin E levels than those fed SBM, suggesting the enhancement of antioxidant capacity of dietary functional additives. For the disease resistance against *Vibrio harveyi* infection, fish fed SBM had the highest cumulative mortality, followed by the FAS and FM groups. Additionally, the biomarkers related to the immune and antioxidant capacities had a positive correlation with the relative abundance of *Paracoccus* and *Pseudomonas*, while liver MDA levels had a negative correlation with the relative abundance of *Pseudomonas* and *Psychrobacter*. Collectively, soymeal replacing 50% of fishmeal suppressed the growth, immunity, antioxidant capacity and disease resistance of Japanese seabass, while dietary supplementation of antioxidants and chelated trace elements could mitigate soymeal-induced adverse effects on growth and disease resistance through the improvement in antioxidant capacity and regulation of gut microbiota.

## 1. Introduction

Japanese seabass (*Lateolabrax japonicus*), as one euryhaline and carnivorous species, has been widely cultured in Eastern Asia due to its commercial value. In recent decades, the anxieties of unsustainable and costly wild-captured forage fish driven by the expansion of aquaculture production has driven scientists to find economical and nutritious fishmeal alternatives. Studies on fishmeal alternatives have been continually conducted in Japanese seabass, including, but not limited to, defatted black soldier fly larvae meal [1], porcine meal [2], animal protein blend [3], canola meal [4], corn gluten meal [5] and cottonseed meal [6] as well as soymeal [7,8,9,10,11,12,13]. Among these fishmeal alternatives, conventional soybean meal and its relevant products have received more focus in Japanese seabass due to its stable production, relatively high digestibility and low market price. However, several obvious disadvantages in conventional soybean meal, such as a deficiency of essential nutrients, poor palatability and the existence of anti-nutritional factors, induced enteritis and metabolic liver disease, have limited its high utilization in aquafeed [6,14,15]. In general, previous studies in Japanese seabass demonstrated that conventional soybean meal replacing more than 50% of fishmeal protein could adversely influence the digestive abilities, immune responses and antioxidant abilities and consequently suppress the growth performance and health of the host [8,9,11,16]. Aiming to optimize soymeal-based diets for Japanese seabass, a range of processing approaches, such as fermentation and gamma-irradiation, have been implemented to partially remove anti-nutritional factors, but replacing more than 50% of fishmeal still induces negative effects on growth performance and health under certain conditions [10,13,16].

Based on this background, to increase the utilization of conventional soybean meal and mitigate its negative influence, expanded evaluations of functional additives on the growth performance and health of fish fed soymeal-based diets are necessary. The administration of functional additives could be achieved alone or in combination. In general, a mixture of functional additives rather than a single substance could provide more effective effects on the growth and health of the host [17,18,19]. Thus, in the current study, a mixture of antioxidants, i.e., ethoxyquin and a 1:1 standardized combination of thymol and carvacrol and chelated trace elements, i.e., Mintrex^®^ Cu, Mn and Zn, was selected to be tested. The ethoxyquin, as a feed preservative, has been used in aquafeed for many years [20]. The 1:1 standardized combination of thymol and carvacrol are essential oils from thyme and oregano extracts that can improve the growth performance, digestive abilities, antioxidant activity and gut microbiota and boost the general health and immune responses of fish under less desirable conditions, such as lower or non-dietary fishmeal diets, stress or disease [21]. Regarding the chelated trace elements, Mintrex^®^ Cu, Mn and Zn not only keep trace elements balanced but could also improve growth performance and antioxidant ability and balance the requirements of essential amino acids [22]. Further, supplemental chelated trace elements may become more necessary due to the existence of antagonists in soybean meal that would bind with divalent cationic trace elements [23,24].

Therefore, this study aimed to evaluate whether functional additives could mitigate the negative effects on growth, immunity, antioxidant capacity and disease resistance against *Vibrio harveyi* infection in Japanese seabass fed diets with conventional soymeal meal replacing 50% of fishmeal.

## 2. Materials and Methods

### 2.1. Fish Husbandry 

This experiment was conducted following the Management Rule of Laboratory Animals (Order No. 676 of the Chinese State Council). The Japanese seabass were purchased from a commercial producer located in Qinzhou, Guangxi, China. Before the feeding trial, all fish were stocked and fed a commercial diet for about two weeks to acclimate to the experimental conditions in floating sea cages (4.0 m × 5.0 m × 5.0 m) in Qinzhou, Guangxi, China. Subsequently, fish with an initial average body weight of 125.6 ± 0.6 g were randomly transported into 12 floating sea cages (1.5 m × 3.0 m × 3.0 m) where each treatment was split into quadruplicate cages, each containing 40 fish. Fish were hand-fed continuously to satiation twice daily (06:00 and 18:00) for 105 days. Regarding the water parameters, the water temperature followed a natural fluctuation ranging from 14 to 26 °C, and the water salinity and dissolved oxygen were above 20‰ and 7 mg L^−1^, respectively, for the entire feeding period.

### 2.2. Diets

Table 1 shows the formulation and nutrient composition of the experimental diets. Specifically, three isonitrogenous (45% crude protein) and isolipidic (11% crude lipid) experimental diets were formulated. One standard reference diet, named FM, was formulated based on the commercial diet for Japanese seabass containing 42% fishmeal. Two experimental diets with (named FAS) and without functional additives (named SBM) were formulated using conventional soybean meal as the only fishmeal alternative with the substitution level at 50%. The formulation of the functional additives was the mixture of ethoxyquin (0.02% Agrado^®^, Columbia), a 1:1 standardized combination of thymol and carvacrol (0.006% NE-150^®^) as well as chelated trace elements (0.0055% Mintrex^®^ Cu, 0.01% Mintrex^®^ Mn and 0.025% Mintrex^®^ Zn) (Novus International Inc., St. Charles, MO, USA). Each diet was produced into proper sizes of floating pellets (4- and 5-mm diameter) using a twin-screwed extruder (EXT50A, Yanggong Machine, Yangzhou, China) and stored at 20 °C until used.

### 2.3. Sample Collection

At the end of the feeding trial, fish were anesthetized with eugenol (1:10,000) before tissue sampling. The fork length and body weight of all fish were recorded, and the plasma samples were obtained from the caudal vein of 3 fish per sea cage, i.e., 12 fish per dietary treatment. Plasma was collected after centrifugation at 2000× *g* for 10 min at 4 °C and frozen in liquid N_2_ and then stored at −80 °C prior to analysis. The livers of fish were collected from 3 fish per cage, i.e., 12 fish per dietary treatment, and then frozen at −80 °C until analysis.

### 2.4. Proximate Composition of Diets and the Whole Body of Fish

Duplicate analyses of the moisture, crude protein and crude lipid as well as the ash of diets and the whole bodies of fish were analyzed according to the standard protocols in our previous descriptions [6].

### 2.5. Respiratory Burst Activity (RB) Assay

The head kidney macrophages of 3 fish per sea cage, i.e., 12 fish per dietary treatment, were isolated according to the previous description [25]. Briefly, the head kidney was collected and cut into small fragments and then washed with Leibovitz-15 medium (L-15, Sigma, St. Louis, MO, USA) containing 100 IU/mL penicillin, 100 IU/mL streptomycin and 2% fetal calf serum (Gibco, Invitrogen, New York, NY, USA). Subsequently, the cell suspensions were prepared by forcing the head kidney to pass through a 100 µ steel mesh. The resultant cell suspensions were enriched by centrifugation at 400× *g* for half an hour at 4 °C using the 51% (*v/v*) Percoll density gradient (Pharmacia, St. Louis, MO, USA). The bands of cells were collected at the 51% interface and then washed twice using the L-15 medium. Cell viability of the head kidney was determined by the trypan blue exclusion method, and the cell density was measured using the hemocytometer. Finally, the L-15 medium was added to adjust the cell concentration (1 × 10^7^ mL^−1^) for analysis.

For the respiratory burst activity analysis, the production of intracellular superoxide anion (O^2−^) was measured using nitroblue tetrazolium (NBT) reduction (Sigma, Louis, MO, USA). A 100 μL cell suspension was stained using 100 μL of 0.3% NBT and 100 μL of 1 mg/mL phorbol 12-myristate 13-acetate (Sigma, Louis, MO, USA) for 40 min, and then the absolute methanol was added to terminate the staining reaction. The 70% methanol was used to wash the cell suspension three times. Subsequently, 120 μL of 2 M KOH and 140 μL of DMSO were added, and then the color was measured at 630 nm using a spectrophotometer (Thermo Fisher Scientific, Waltham, MA, USA) with KOH/DMSO as the blank.

### 2.6. Serum Alternative Complement Pathway (ACP) Activities

The serum ACP activities were measured as previously described [26]. Briefly, volumes of diluted serum ranging from 0.1 to 0.25 mL were dispensed into 5 mL test tubes. The total reaction volumes were increased up to 0.25 mL using a barbitone buffer with a mixture of thyleneglycolbis (2-aminoethoxy)-tetraacetic acid and Mg^2+^, and then 0.1 mL of rabbit red blood cells were added to each test tube. After incubation for 1.5 h at 22 °C, 3.15 mL of 0.9% sodium chloride was added to each test tube. Subsequently, the test tubes were centrifuged at 1000× *g* for 5 min at 4 °C to remove unlysed rabbit red blood cells. The optical density of the supernatant was measured at 414 nm using the spectrophotometer (Thermo Fisher Scientific, Waltham, MA, USA). The serum reaction volume producing 50% hemolysis (ACH50) was measured and recorded.

### 2.7. Serum Antioxidant Enzyme Assay

The serum copper-zinc superoxide dismutase (Cu-Zn SOD) activities were measured spectrophotochemically by the ferricytochrome C method using xanthine/xanthine oxidase as the source of superoxide radicals according to the protocol of the commercial assay kit (No 19160, Sigma, Louis, MO, USA). The results were expressed as Cu-Zn SOD unit mL^−1^ and the unit was defined as the amount of enzyme necessary to create a 50% inhibition of the ferricytochrome C reduction rate measured at 550 nm.

The serum catalase (CAT) activities were determined according to the standard protocol [27]. The initial rate of hydrogen peroxide decomposition was measured, and one unit of serum CAT activity was defined as the amount of enzyme that catalyzes the decomposition of 1.0 µmol of hydrogen peroxide per second.

The serum glutathione peroxidase (GPX) activities were measured according to the previous protocol [28]. Briefly, the serum GPX activities were measured by quantifying the rate of hydrogen-peroxide-induced oxidation of reduced glutathione to oxidized glutathione. The reaction volume of the yellow product had absorbance at 412 nm that could be formed as glutathione reacted with dithiobisnitrobenzoic acid.

### 2.8. Liver Malondialdehyde (MDA) Levels

The liver MDA levels were measured using the thiobarbituric acid method according to the standard protocol of the commercial kit (MDA detection kits, Jincheng Bioengineering Ltd., Nanjing, China). The MDA could form a red adduct with thiobarbituric acid (TBA) that could be measured at the wavelength of 532 nm by reacting with TBA to form a stable chromophoric production. The MDA was calculated in nmol/mg using the calibration curve of the standard addition method and then expressed as nanomoles per milligram of protein in the liver (nmol/mg protein). The liver protein levels were measured according to the standard protocol of the commercial assay kits (Nanjing Jiancheng Institute, Nanjing, China).

### 2.9. Liver Vitamin E Concentrations 

The liver vitamin E concentrations were measured using reverse-phase high-performance liquid chromatography based on the method in one previous study [29]. Briefly, the mobile phase included a mixture of 95% methanol and 5% water. The solvent was filtered through a 0.45 μm filter and then degassed. The pump flow rate was 1.0 mL/min. The standard solution of DL-α-tocopheryl acetate was prepared in high-performance liquid-chromatography-grade methanol and then stored at 4 °C in the dark. Subsequently, 20 microliters of the prepared sample were injected into the high-performance liquid chromatography system and then the C18 μ bondapack column was used. The absorbance of the reaction volume was measured using the UV detector at 280 nm (Waters, Taunton, UK).

### 2.10. Liver Scavenging Rate of Hydroxyl Radical (SROH)

The liver scavenging rate of ^•^OH was measured according to the 1, 10-phenanthroline-Fe^2+^ -Fenton reaction method as previously described [30]. Briefly, a 0.5 mL solution of phenanthrene anhydrous ethanol (0.75 mmol/L) was added and mixed with 1 mL of 0.2 mol/L phosphate buffer solution (PBS, pH 7.40) and 0.5 mL of distilled water. Next, the solution was mixed with 0.5 mL of 0.75 mmol/L ferrous sulfate solution (FeSO_4_) and then added with 0.5 mL of 0.01% hydrogen peroxide (H_2_O_2_). The reaction mixture was incubated at 37 °C for 60 min in a water bath, and its absorbance was measured at 536 nm. The SROH was expressed as: scavenged rate (%) = (A_1_ − A_0_)/(A_s_ − A_0_) ×100, where A_1_ was the absorbance of the sample, A_s_ was the absorbance in the presence of a positive control using distilled water, and A_0_ was the absorbance in the presence of a negative control using H_2_O_2_.

### 2.11. Challenge Test

The *Vibrio harveyi* strain (reference sequence: NZ-JH720471.1, NCBI) was originally isolated from infected Japanese seabass. The 10-day LD50 (*V. harveyi* dose that killed 50% of the test Japanese seabass) was measured by intraperitoneal injection of 60 fish with graded doses of *V. harveyi* (10^6^, 10^7^, 10^8^ and 10^9^ CFU/fish) at 20 °C. The results showed that the LD50 was 10^8^ CFU/fish [31]. At the termination of the feeding experiment, 15 fish from each cage were collected and transported into 12 tanks with an independent culture system in an indoor facility and then injected intraperitoneally with 0.2 mL of PBS containing 2 × 10^8^ live *V. harveyi* from a 24 h culture at 25 °C. Subsequently, the cumulative mortality was calculated for 10 days.

### 2.12. Calculations and Statistical Methods

Survival rate (%) = 100 × number of final fish/number of initial fish

Weight gain rate (WG, %) = 100 × ((final body weight − initial body weight)/initial body weight)

Feed intake (FI, %/day) = 100 × feed consumption/((final body weight + initial body weight + weight of dead fish)/2)/days

Feed conversion ratio (FCR) = feed consumed (dry weight)/weight gain

Condition factor (CF, g/cm^3^) = 100 × body weight/body length^3^

Hepaticsomatic index (HIS, %) = 100 × liver weight/body weight

Viscerasomatic index (VSI, %) = 100 × viscera weight/body weight

Except for the figure balloon plot, all other figures and data analyses were conducted using GraphPad Prism 9 (GraphPad Software, San Diego, CA, USA). Data were evaluated for homogeneity of variances and normality using the Shapiro–Wilk and Barlett’s test, respectively. A one-way ANOVA followed Tukey–Kramer HSD multiple comparisons were conducted to compare the mean values among the dietary treatments. All results were presented as means ± S.E.M (standard errors of the mean). The level of significant differences was set at *p* < 0.05.

The Pearson correlation between the gut microbiota and biomarkers in this study from the same sea cage was analyzed using GraphPad Prism 9. The balloon plot was made by TBtools [32] based on the statistical results of the Pearson correlation. Values marked with white symbol “*” are significant correlations (*p* < 0.05), and “**” are very significant correlations (*p* < 0.01).

## 3. Results

### 3.1. Growth Performance

**Figure 1:**
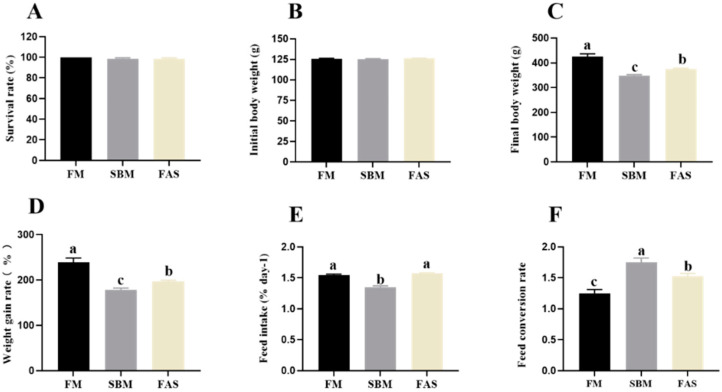
Survival rate (**A**) and growth performance (**B**–**F**) of Japanese seabass fed diets using soybean meal as fishmeal alternative with or without functional additives. Data are presented as means ± SEM. Bars sharing the same letters are not significantly different (*p* > 0.05, *n* = 4).

In our study, the survival rate of Japanese seabass averaged above 98.75% regardless of the experimental diets (*p* > 0.05, Figure 1A). Fish fed FM showed the highest growth performance, estimated as the final body weight and WG, compared to those fed soymeal-based diets (*p* < 0.05, Figure 1B–D), and fish fed FAS had higher growth performance than those fed SBM (*p* < 0.05, Figure 1B–D).

A lower FI was observed in fish fed SBM compared to those in the FM and FAS groups (*p* < 0.05, Figure 1E), and no clear difference was observed the between FM and FAS groups (*p* > 0.05, Figure 1E). Regarding FCR, fish fed SBM showed the highest value, followed by the FAS and FM groups (*p* < 0.05, Figure 1F).

### 3.2. Body Indexes

**Figure 2:**
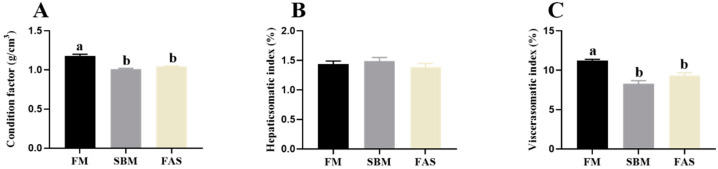
Body indexes of Japanese seabass fed diets using soybean meal as fishmeal alternative with or without functional additives. (**A**) Condition factor. (**B**) Hepaticsomatic index. (**C**) Viscerasomatic index. Data are presented as means ± SEM. Bars sharing the same letters are not significantly different (*p* > 0.05, *n* = 12).

Similar to the growth performance, the highest CF and VSI values were observed in fish fed FM compared to those fed soymeal-based diets (*p* < 0.05, Figure 2A,C) and these values were not significantly affected by dietary functional additives (*p* > 0.05, Figure 2A,C). No clear difference was found in the HSI of fish among the dietary treatments (*p* > 0.05, Figure 2B).

### 3.3. Proximate Composition of the Whole Body of Fish

Fish fed FM had significantly lower whole-body moisture than those in fish fed SBM and FAS (*p* < 0.05, Table 2), whereas there was not a significant difference in whole-body moisture between fish fed SBM and FAS (*p* > 0.05, Table 2). The whole-body crude lipid showed the opposite trend among dietary treatments, with the highest value in fish fed FM (*p* < 0.05, Table 2). Regarding the whole-body crude protein and ash, there were no significant differences among the dietary treatments (*p* > 0.05, Table 2).

### 3.4. Immune Parameters

**Figure 3:**
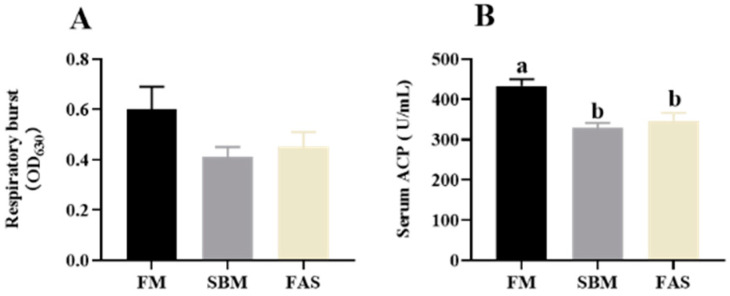
Immune responses of Japanese seabass fed diets using soybean meal as fishmeal alternative with or without functional additives. (**A**) Respiratory burst activity of the head kidney macrophages. (**B**) Serum alternative complement pathway (ACP) activity. Data are presented as means ± SEM. Bars sharing the same letters are not significantly different (*p* > 0.05, *n* = 12).

Fish fed FM had higher respiratory burst activities in macrophages of head kidneys than those fed soymeal-based diets but there was no statistical difference among the dietary treatments (*p* > 0.05, Figure 3A). The highest serum ACP activities were observed in fish fed FM compared to those fed soybean meal diets, i.e., the SBM and FAS groups (*p* < 0.05, Figure 3B), whereas the ACP activities were not significantly influenced by dietary functional additives (*p* > 0.05, Figure 3B).

### 3.5. Antioxidant Capacities

**Figure 4:**
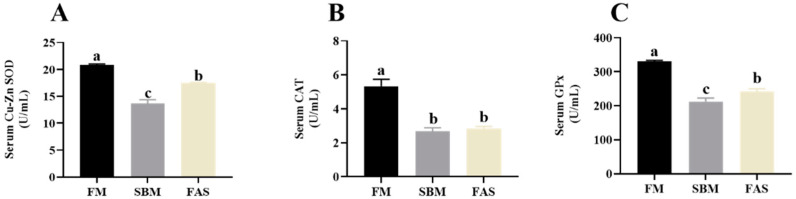
Antioxidant capacity in serum of Japanese seabass fed diets using soybean meal as fishmeal alternative with or without functional additives. (**A**) Serum copper-zinc superoxide dismutase (Cu-Zu SOD) activity. (**B**) Serum catalase (CAT) activity. (**C**) Serum glutathione peroxidase (GPx) activity. Data are presented as means ± SEM. Bars sharing the same letters are not significantly different (*p* > 0.05, *n* = 12).

The results showed that fish fed FM revealed higher serum Cu-Zn SOD, CAT and GPx activities than those fed soymeal-based diets (*p* > 0.05, Figure 4). Of note, fish fed the soymeal-based diet with functional additives, i.e., the FAS group, had higher serum Cu-Zn SOD and GPx activities than those fed a similar diet without functional additives, i.e., the SBM group (*p* < 0.05, Figure 4A,C).

**Figure 5:**
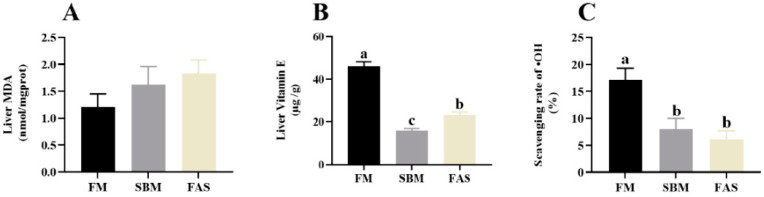
Antioxidant capacity in the liver of Japanese seabass fed diets using soybean meal as fishmeal alternative with or without functional additives. (**A**) Liver malondialdehyde (MDA) level. (**B**) Liver Vitamin E level. (**C**) Liver scavenging rate of hydroxyl radical (^•^OH). Data are presented as means ± SEM. Bars sharing the same letters are not significantly different (*p* > 0.05, *n* = 12).

Although there was an increasing trend in liver MDA levels from FM to FAS, no significant statistical difference was observed among the dietary treatments (*p* > 0.05, Figure 5A). Like the serum antioxidant capacities, the vitamin E levels and scavenging rate of ^•^OH in the liver had higher values in fish fed FM compared to fish fed SBM and FAS (*p* < 0.05, Figure 5B,C). Regarding the effects of dietary functional additives, fish fed FAS significantly increased liver vitamin E levels compared to those fed SBM (*p* < 0.05, Figure 5B). 

### 3.6. Challenge Test

**Figure 6:**
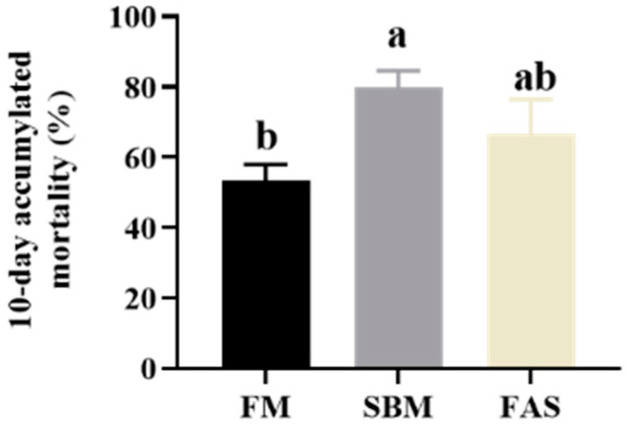
Ten-day accumulated mortality in Vibrio harveyi challenge test of Japanese seabass fed diets using soybean meal as fishmeal alternative with or without functional additives. Data are presented as means ± SEM. Bars sharing the same letters are not significantly different (*p* > 0.05, *n* = 4).

After the 10-day challenge test, fish fed the SBM diet had the highest cumulative mortality rate of V. harveyi infection (80%), followed by fish fed the FAS (66.7%) and FM (53.3%) diets (*p* < 0.05, Figure 6).

### 3.7. Pearson Correlation between Gut Microbiota and Host Responses

To help readers to interpret the Pearson correlation results we present here, results of gut microbiota that have been reported elsewhere [7] are briefly summarized as the following: Gut contents from the whole intestine of three fish per sea cage were collected and pooled for microbiota analysis. Ten genera (*Halomonas*, *Shewanella*, *Paracoccus*, *Methylobacterium*, *Ochrobactrum*, *Stappia*, *Corynebacterium*, *Pseudomonas*, *Acinetobacter* and *Psychrobacter)* strongly dominated the gut microbiota of Japanese seabass, accounting for about 70% of total abundance. Further, dietary functional additives could mitigate soymeal-induced enteritis, whereas the composition of gut microbiota was not clearly affected by dietary treatments according to the alpha and beta diversity.

**Figure 7:**
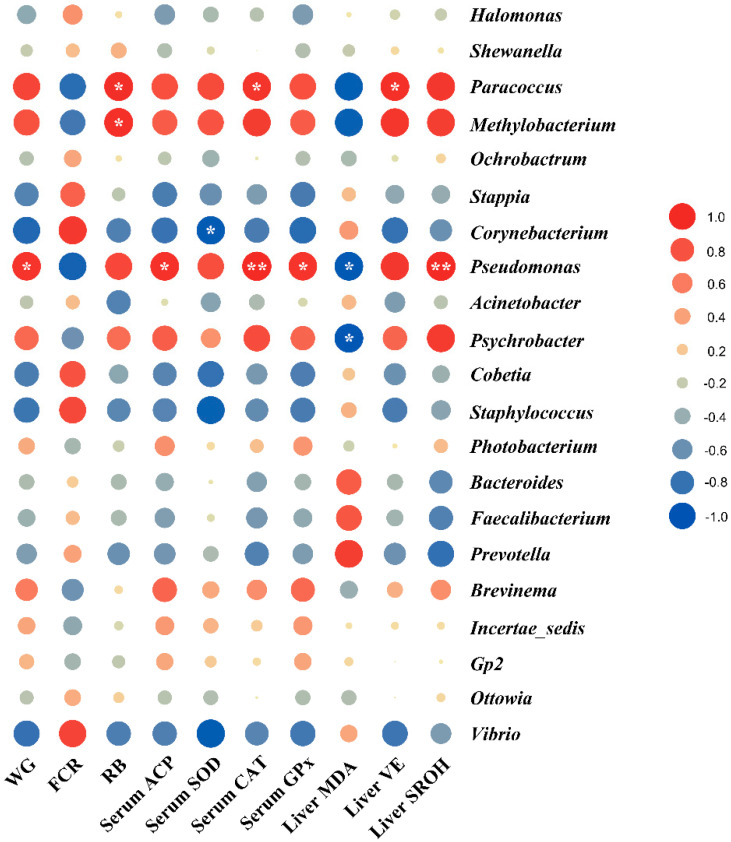
The Pearson correlation between gut microbiota and host responses. Circles in the balloon plot denoting the direction of the association are colored red (positive) or blue (negative) and are overlaid with the white symbol “*” indicating a significant correlation. Values marked with a white symbol “*” are significant correlations (*p* < 0.05), and “**” are very significant correlations (*p* < 0.01). WG: weight gain rate; FCR: feed conversion ratio; RB: respiratory burst activity; ACP: alternative complement pathway; SOD: copper-zinc superoxide dismutase; CAT: catalase; GPx: glutathione peroxidase; MDA: malondialdehyde; VE: Vitamin E; SROH: scavenging rate of hydroxyl radical.

For the results of the Pearson correlation analysis, the respiratory burst activity in macrophages of head kidneys, serum CAT and liver vitamin E had strong positive correlations with the relative abundance of *Paracoccus*. Moreover, the respiratory burst activity in macrophages of head kidneys had a positive correlation with the relative abundance of *Methylobacterium*. Additionally, weight gain, serum ACP, CAT and GPx as well as liver SROH had strong positive correlations with the relative abundance of *Pseudomonas*, while the liver MDA had a negative correlation with the relative abundance of *Pseudomonas* and *Psychrobacter* (*p* < 0.05, Figure 7).

## 4. Discussion

### 4.1. Growth Performance and Body Composition

Fish fed the soymeal-based diets with or without functional additives, i.e., the SBM and FAS groups, suppressed the growth performance in the present study, indicating that Japanese seabass cannot tolerate conventional dietary soybean meal as a fish meal substitute at 50%. Overall, previous studies concluded that the optimal replacement level of fishmeal with conventional soybean meal is highly variable for carnivorous fish species. For example, similar to our findings, the optimal replacement level should be less than 50% in some carnivorous fish species, including, but not limited to, Atlantic salmon, *Salmo salar* (30%) [33], Chinese sucker, *Myxocyprinus asiaticus* (30%) [34], European sea bass, *Dicentrarchus labrax* (25%) [35], Japanese flounder, *Paralichthys olivaceus* (24%) [36], spotted rose snapper, *Lutjanus guttatus* (20%) [37], and turbot, *Scophthalmus maximus* L. (40%) [38]. However, certain carnivorous fish species could tolerate conventional dietary soybean meal replacing more than 80% of fish meal without negative effects on growth performance, such as hybrid striped bass, *Morone chrysops* × *M. saxatilis* [39], and largemouth bass, *Micropterus salmoides* [40].

Regarding Japanese seabass, studies related to the use of conventional soybean meal as a fishmeal alternative have been widely reported, but the results varied greatly between studies due to the differences in fish size, feed formulation, feeding strategy and rearing conditions. Some previous studies supported our findings [8,9,13], while other studies found that replacing up to 50% of fishmeal did not influence the growth performance [11,12]. Generally, the three causes of soymeal-based diet inducing inferior growth performance may occur alone or together: (1) low palatability; (2) anti-nutritional factors; and (3) a shortage of essential nutrients. 

In our study, a lower FI was observed in fish fed SBM compared to those fed FM, indicating a poor palatability of the SBM diet, as the palatability is an important driver of FI. Further, various anti-nutritional factors in soybean meal, for example, saponin, phytic acid, lectin and protease inhibitor, have been widely reported to decrease palatability, induce enteritis and consequently suppress growth performance [15,33], as also supported by our study reported elsewhere [7]. To eliminate certain anti-nutritional factors of soybean meal, various processing approaches, such as fermentation and gamma-irradiation, have been implemented. These processed soybean meal products could successfully replace more than 50% of fishmeal without an adverse influence on the growth performance and digestibility of Japanese seabass [13]. Furthermore, lacking essential amino acids, e.g., lysine and methionine, and nutrients related to bile acid synthesis, e.g., cholesterol and taurine, has been reported to limit the growth performance of fish [6,34]. In terms of the former, methionine hydroxy calcium, blood meal and Mintrex^®^ chelated trace elements have been supplemented to dietary treatments to ensure the requirement of methionine and lysine in the present study. The dietary methionine (0.96–1.02% of dry diet) and lysine (2.66–2.74% of dry diet) were all above the requirement, i.e., 0.9% and 2.6% of the dry diet, respectively, estimated by Mai et al. [41]. Hence, the deficiency of methionine and lysine might not be the main reasons for the inferior growth performance in our study. Regarding the nutrients related to bile acid synthesis, since the level of these nutrients and bile acid metabolism were not observed in this study, complementary studies warrant further investigation.

Notably, fish fed FAS showed an FI similar to those fed FM and higher than those fed the SBM diet. Meanwhile, a higher growth performance was observed in FAS compared to the SBM group. These findings suggest that dietary functional additives could increase FI and improve growth performance. In addition, fish fed the FAS diet exhibited remarkably higher growth performance and FCR, possibly suggesting the functional additives herein might partially eliminate the negative influence on growth performance caused by antinutritional factors through the improvement in digestibility, immune response and antioxidant capacity [21,22], as discussed below.

Higher values of CF and VSI as well as whole-body crude lipid content were found in fish fed FM compared to soymeal-based diets, which is in line with previous studies in Japanese seabass [11,12,13,16]. Of note, the increase in VSI and whole-body crude lipid content could be at least partially responsible for the increase in growth performance, possibly due to the increase in feed digestibility and digestive enzyme activity [11,13].

### 4.2. Immunity

Nutritional factors play an important role in immune responses in fish [42]. Previous studies have found that the administration of high levels of soybean meal suppressed immune responses in turbot [43] and red sea bream [44] as well as Japanese seabass [16]. Likewise, fish fed soymeal-based diets suppressed the serum ACP activities in the current study. Further, the respiratory burst activity in macrophages of head kidneys showed a similar picture as serum ACP but there were no statistical differences. Reductions in complement activities in soymeal-based diets were also found in one previous study in Japanese seabass that replaced 80% of fishmeal with soybean meal, causing significant decreases in complement C3 activities [16]. The suppression of immune responses is likely to be the result of the presence of anti-nutritional factors and antigens in soybean meal that damage the immune system in fish [15,16]. On the other hand, fish fed SBM had the highest cumulative mortality of *Vibrio harveyi* infection compared to soymeal-based diets. This is in line with the previous studies in yellowtail, *Seriola quinqueradiata,* where a high dietary level of soymeal suppressed immune functions and increased host–pathogen susceptibility [45].

Additionally, in our study, supplementing these functional additives in the SBM group might boost immune responses of Japanese seabass in the FAS group, as concluded in one previous review [21]. Results of decreased cumulative mortality in the current study and induced enteritis [7] in the FAS group could further confirm that these selected functional dietary additives could partially mitigate the suppression of immunity.

### 4.3. Antioxidant Capacity

The antioxidant capacity is an important indicator of the health status of fish. Oxidative stress occurs in fish when the reactive oxygen species (ROS) generation rate exceeds that of their removal abilities [46]. On one hand, a wide range of antioxidant substances, such as Vitamin E, play the first line of defense against the adverse effects of oxidative stress and other free radicals [47]. The FM-fed group was found to increase the level of liver Vitamin E compared to those fed soymeal-based diets in our study, suggesting dietary soybean meal reduced the number of antioxidant substances and suppressed the antioxidant capacity, as further supported by the results of the scavenging rate of ^•^OH in the present study. This is also consistent with the previous claim that soybean meal could affect antioxidative defense due to the existence of anti-nutritional components in a plant-based diet, such as tocopherols [48]. On the other hand, various radical scavenging antioxidant enzymes, for example, Cu-Zn SOD, CAT and GPx, play the second line of defense to prevent the cascade of oxidant reactions and serve as significant biomarkers for the immune and antioxidant status of the host [46]. In our study, soymeal-based groups exhibited lower levels of these antioxidant enzyme activities in serum compared to the FM group. These findings are paralleled by previous studies in turbot [43] and Japanese seabass [16], further suggesting dietary soybean meal suppressed antioxidant capacity. Moreover, the MDA is the final product of lipid peroxidation, which could be a valuable biomarker for endogenous oxidative damage. In this study, high liver MDA levels were observed in the FM group, but there was no significant statistical difference. Collectively, these results in our study further support the theory that soybean meal replacing 50% of fishmeal led to a significant reduction in antioxidant capacities.

One of the key purposes of dietary functional additives, especially natural products, is to improve the antioxidant capacities of fish. The application of thymol and carvacrol as natural antioxidant feed additives in aquafeed plays an important role in improving antioxidant activities and health [21]. Moreover, dietary chelated trace minerals (Cu, Mn and Zn) rather than inorganic minerals could also improve antioxidant ability, such as Cu-Zn SOD activities, which have been widely reported in fish and shrimp [22,23,24]. Likewise, in our study fish fed FAS had higher serum Cu-Zn SOD and GPx activities as well as liver vitamin E levels than those fed SBM, indicating that dietary functional additives could significantly enhance antioxidant capacities via the increase in antioxidant substance levels and antioxidant enzyme activities. The increase in antioxidant activity could be at least partially responsible for the improvement in growth performance and disease resistance against *Vibrio harveyi* infection in this study.

### 4.4. Associations between Gut Microbiota and Host Responses

Most fish microbiota studies published so far are descriptive studies on the taxonomic composition and its changes under different experimental conditions. To identify the major microbial clades that benefit fish health and welfare, characterizing the associations between gut microbiota and host responses is a necessary step [49,50,51]. In the current study, we found that the relative abundance of the genus *Pseudomonas* showed a strong positive correlation with the biomarkers related to the immune and antioxidant capacities, while there was a negative correlation with liver MDA levels, suggesting dietary treatments could shape key intestinal microbial clades and thereby regulate host immune and antioxidant functions. However, the literature reporting gut microbiota and its correlation with immunity and antioxidant capacities in Japanese seabass is still limited so far, and the mechanisms behind these correlations remain unknown. Of note, the *Pseudomonas* has been widely found in the gut microbiota of Atlantic salmon using the culturing and molecular approaches [51,52], and it was found that its relative abundance was negatively correlated with the distal intestine somatic index [51]. These findings call for more investigation to increase the information on the associations between *Pseudomonas*, immune responses and antioxidant capacities in Japanese seabass.

## 5. Conclusions

The findings in the present study showed that Japanese seabass fed the diet with conventional soybean meal replacing 50% of fishmeal exhibited suppressed growth performance, immunities, antioxidant capacities and disease resistance against *Vibrio harveyi* infection. Moreover, supplementation of a mixture of antioxidants (ethoxyquin, thymol and carvacrol) and chelated trace elements (Cu, Mn and Zn) in the diet with conventional soybean meal replacing 50% of fishmeal could mitigate soymeal-induced adverse effects on growth and disease resistance via improvements in the antioxidant capacities and gut microbiota.

## Figures and Tables

**Table 1:** Formulation and nutrient composition of the experimental diets.

Ingredients (% Dry Matter)	FM	SBM	FAS
Fishmeal ^1^	42	21	21
Conventional soybean meal ^1^	12	30	30
Wheat ^1^	25.8	22.1	22.1
Peanut meal ^1^	10	10	10
Squid meal ^1^	2.5	3.5	3.5
Spray blood meal ^1^		2.8	2.8
Soy protein concentrate ^1^		2	2
Fish oil	4	5.5	5.5
Vitamin and mineral premix	1	1	1
Lecithin	1	1	1
Vitamin C	0.1	0.1	0.1
Calcium propionic acid	0.1	0.1	0.1
Choline	0.5	0.5	0.5
Monocalcium phosphate	1	1	1
MeraMet ^2^		0.26	0.26
Threonine ^2^		0.1	0.1
Ethoxyquin	0.02	0.02	0.02
Functional additives ^3^			0.0665
Proximate analysis			
Crude protein	45.2	45.1	44.9
Crude lipid	11.6	11.4	11.2
Lysine	2.74	2.66	2.68
Methionine	0.96	0.99	1.02

^1^ These ingredients were purchased from Great Seven Bio-Tech, Qingdao, China. Fish meal: protein 63.90, lipid 10.15; Squid meal: protein 56.67, lipid 28.85; Peanut meal: protein 55.99, lipid 6.46; Spray blood meal: protein 98.92, lipid 0.99; Soybean meal: protein 54.51, lipid 1.48; Soy protein concentrate: protein 73.32, lipid 1.04. ^2^ MeraMet is methionine hydroxy calcium. Methionine, threonine and lysine were satisfied for fish in all the treatments. ^3^ The functional additives were from Novus International Inc., St. Charles, MO, USA.

**Table 2:** The body composition in Japanese sea bass fed functional additives.

(% Wet Weight)	FM	SBM	FAS
Moisture	67.0 ± 0.9 ^b^	71.6 ± 0.4 ^a^	71.1 ± 0.6 ^a^
Crude lipid	11.2 ± 0.7 ^a^	7.0 ± 0.4 ^b^	8.0 ± 0.3 ^b^
Crude protein	16.9 ± 0.7	16.8 ± 0.1	16.5 ± 0.4
Ash	4.4 ± 0.2	5.2 ± 0.1	5.0 ± 0.2

Note: values are represented as means ± S.E.M (*n* = 12); values in the same row with the same superscripts are not significantly different (*p* > 0.05).

## Data Availability

The data presented in this study are available on request from the corresponding author. The data are not publicly available due to containing information that could compromise the privacy of research participants.
